# Integrating Dental Healthcare into Primary Health Care through an implementation model in two districts of Karnataka in India: An implementation research study protocol

**DOI:** 10.1136/bmjopen-2025-111996

**Published:** 2026-06-10

**Authors:** Lathadevi Chilgod, Ramya Krishna B M, Raveena Raveendran, Pragati B Hebbar

**Affiliations:** 1 Centre for Health Systems, Institute of Public Health Bengaluru, Bengaluru, India; 2 T T Narasimhan School of Advanced Studies, Institute of Public Health Bengaluru, Bengaluru, India

**Keywords:** Primary Health Care, Dentistry, Health Equity, Implementation Science, Health Services, Delivery of Health Care, Integrated

## Abstract

**Abstract:**

**Introduction:**

Despite progress in primary care, access to oral health remains limited in India. Integrating oral health into primary care can improve oral health outcomes for communities, especially in rural areas. This study aims to develop an implementation model for integrating dental healthcare into primary healthcare systems in Chamarajanagar and Mysuru districts of Karnataka.

**Methods and analysis:**

A mixed-methods implementation research approach will be adopted for this study, comprising three phases: formative, pilot and implementation and evaluation.

A mixed-methods study at the district level will be conducted to capture diverse perspectives and characterise inequalities in oral healthcare access and literacy levels across urban, rural and tribal areas. Oral health literacy surveys, facility preparedness checklists and in-depth interviews will be conducted. With a consultative approach involving stakeholders, we ensure their input in the design and pilot the integrated interventions to build a replicable model for integrating dental health services into primary healthcare.

Through this structured approach, the study seeks to improve overall oral health outcomes and achieve sustainable improvements in oral health at both community and system levels.

**Ethics and dissemination:**

This study has been approved by the Institutional Ethics Committee at the Institute of Public Health Bengaluru (IPH/23-24/E/374). Findings will be disseminated through workshops, presentations and publications in peer-reviewed journals.

**STRENGTHS AND LIMITATIONS OF THIS STUDY**

- This study characterises oral health inequalities across urban, rural and tribal communities.
- The co-design, pilot and implementation of an integration model for oral healthcare in partnership with health system actors ensures that the approach is context-specific, participatory and sustainable.
- This study assesses the effectiveness of the integrated oral health solution at the district level.
- The study may be subject to variability in the real-world settings, such as changes in healthcare providers or policies that could influence implementation.
- Data quality for monitoring and evaluation is dependent on the accuracy and consistency of records maintained at primary healthcare centres, taluk hospitals and district hospitals.

## Introduction

 Oral health is a major global health concern, with 45% of the world’s population affected by dental diseases, particularly in low- and middle-income countries (LMICs).^1^,^4^ In India, where up to 75% of the population suffers from dental diseases, several challenges exacerbate the situation, which includes inadequate healthcare-seeking behaviours, insufficient national data on oral health indicators, a shortage of public sector dentists and the dominance of private dental services with high out-of-pocket expenses.^5^,^8^ The poor infrastructure in many regions, coupled with the widespread prevalence of harmful habits such as smoking and smokeless tobacco use, continues to impede progress in addressing oral health issues and complicates efforts to improve outcomes.^9^,^11^

The Dental Council of India (DCI) reports similar data with dental caries affecting approximately one in every two children, underscoring a pervasive and insufficiently addressed public health concern.^12^ The limited availability of public sector dentists and an inequitable allocation of resources in urban areas results in significant gaps in oral healthcare delivery.^13 14^ The high costs of private dental services render treatment inaccessible for a substantial segment of the population, particularly those residing in underserved areas.^14^,^16^

Addressing these challenges of oral health inequities requires innovative solutions, including the adoption of integrated models of care which offer insights into improving oral healthcare accessibility.^17^ Various integrated models, successfully implemented in different contexts, offer valuable insights for improving oral health systems.^13^ For instance, workforce mix strategies in oral healthcare, such as the collaboration among diverse health professionals with community and support groups, including dentists, physicians, nurses, Auxiliary Nurse Midwives, Accredited Social Health Activists (ASHAs), nutritionists and social workers, including the dental home model.^18^,^21^ Other strategies, such as coordinated care, co-locating dental hygienists with medical teams and integrating telehealth systems like the Virtual dental home, further enhance accessibility and continuity of care.^22 23^

Additionally, interprofessional education, coordinated patient care services, closed-loop referral systems and the closer integration of medical and dental providers, as well as alternative approaches like accountable care organisations, have proven effective in fostering comprehensive care delivery.^18 22 24^ Some population-based models emphasise the application of core oral health clinical competencies in primary care settings, including risk assessment, oral health evaluation, preventive interventions, communication and education and interprofessional collaborative practice in primary care settings to address structural barriers and develop interoperable infrastructures.^25^ Such approaches are now central to the current World Health Organization (WHO) global strategy for decentralising dental services through the primary healthcare workforce. Despite the success of these models elsewhere, progress in developing and implementing such frameworks within India has been limited. The Government of India has made efforts to address disparities in oral healthcare through initiatives like the National Oral Health Policy and the incorporation of basic oral care services within Ayushman Arogya Mandirs (AAMs), previously called Ayushman Bharat Health and Wellness Centres.^5 26 27^ However, achieving equitable access to oral healthcare remains a significant challenge; despite improvements in primary healthcare systems, access to oral health services remains restricted.^13 28^ A promising solution lies in integrating oral health into comprehensive primary healthcare policies and public health programmes.^17 29^ One of the six guiding principles of the global strategy on oral health also emphasises incorporating oral health into primary healthcare alongside life course-specific interventions and leveraging digital technologies for oral health.^30^

While some studies underscore the challenges and solutions in integrating oral health into primary care, focusing on enhancing health outcomes and reducing disparities, they emphasise that success depends heavily on structured training for non-dental providers and the establishment of clear referral pathways.^30^,^32^ Key enablers include supportive policies, resource allocation, interprofessional education, collaboration, strategic leadership and close proximity of medical and dental services.^19 29^ Common barriers include a lack of healthcare policies, inadequate interdisciplinary training and increased work demands shaped by contextual and individual factors.^33^ Discipline-specific barriers, such as the perception of oral health as non-critical and limited awareness of its broader health impacts, contribute to its low priority on the political agenda.^34^ The lack of competency among primary care providers at the clinical level further complicates integration efforts.^24^

To address these gaps, innovative approaches are needed to design and implement solutions tailored to specific contexts, particularly in regions with diverse and underserved populations.

The public healthcare system in India is structured as a three-tier hierarchy designed to deliver primary, secondary and tertiary care, operating alongside an extensive private sector. Primary care is the first point of contact, delivered through a network of AAMs and primary health centres (PHCs), former being the grassroot level centre for the community and the latter typically led by an administrative medical officer (MO). Secondary care is provided through community health centres and Taluk Hospitals led by Taluk health officers (THOs), while tertiary care is managed via District Hospitals and Medical Colleges. The Dental Healthcare Access Research and Integration into Primary healthcare in Karnataka (DHARANI) study is situated within this framework across two districts in Karnataka: Mysuru (specifically T. Narasipura taluk) and Chamarajanagar (Kollegala taluk). These taluks represent a diverse demographic mix of urban, rural and tribal populations, served by a total of 22 PHCs (14 in T. Narasipura and 8 in Kollegala) along with their constituent AAMs. Despite this infrastructure, oral health services remain largely ‘dentist-centred’ and concentrated in urban private hospitals, leaving the primary level with significant service gaps. The DHARANI study aims to develop and pilot an implementation model for integrating equitable dental healthcare into primary healthcare in collaboration with health system actors in these two districts (Mysuru and Chamarajanagar) of Karnataka.

The research has three objectives:

1. To characterise inequalities and describe oral healthcare-seeking practices among heterogeneous communities residing in urban, rural and tribal areas of two districts of southern India.
2. To co-design and pilot integrated primary and oral health intervention with health system actors.
3. To build an implementation model for an integrated oral health solution at the district level.

## Methods and analysis

### Study settings

**Figure 1:**
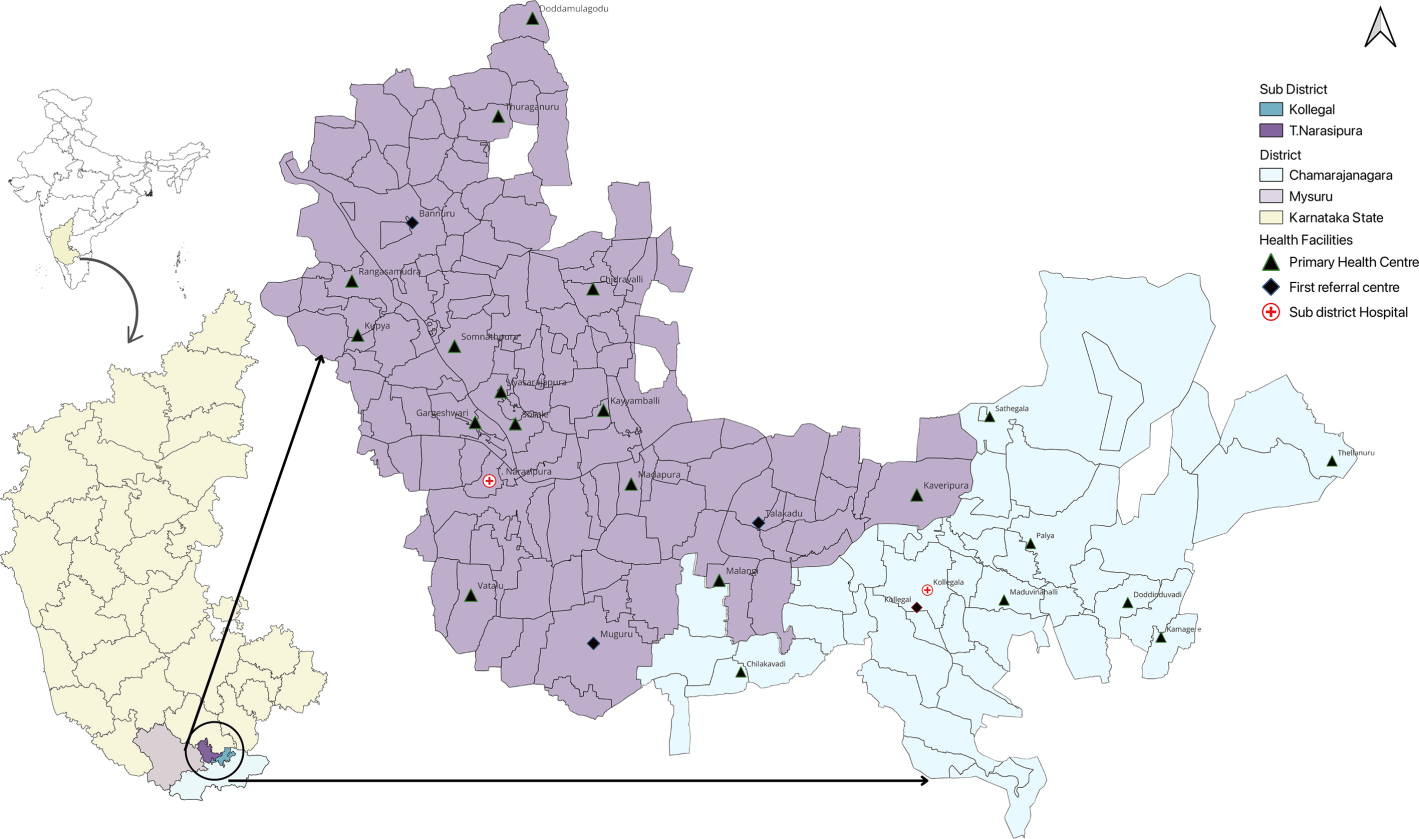
A map showing the two study sites created using QGIS. Data sources: India base geography layer retrieved from geoBoundaries (https://www.geoboundaries.org/); Karnataka state and district layers retrieved from K-GIS (https://kgis.ksrsac.in/kgis/downloads.aspx). KGIS, Karnataka Geographic Information System; QGIS, Quantum Geographic Information System.

The study will take place in two districts of southern Karnataka, India: Chamarajanagar and Mysuru. Within these districts, one block from each, Kollegal taluk in Chamarajanagar and T. Narasipura taluk in Mysuru will be purposefully chosen (figure 1).

## Study design—mixed methods

This study employs an implementation research approach, using mixed-methods organised into three key phases: (I) the formative phase, (II) the pilot and implementation phase and (III) the evaluation phase. The overarching framework for the study is based on the Medical Research Council (MRC) guidance for developing and evaluating complex interventions.^35^ To guide the integration process, the study adopts a modified version of Valentijn *et al*’s integration model that supports the alignment and coordination of healthcare efforts across multiple system levels.^36^

### Phase-wise description

#### Phase I. formative phase

The formative phase seeks to examine inequalities and explore oral healthcare-seeking behaviours among diverse communities residing in urban, rural and tribal regions of Chamarajanagar and Mysuru districts. A mixed-methods approach will be employed, incorporating facility preparedness checklists, oral health literacy surveys and in-depth interviews (IDIs). The facility preparedness checklist is designed to assess the capacity of PHCs to effectively integrate oral health services. The oral health literacy survey, adapted from WHO guidelines, will evaluate the population’s needs, knowledge and awareness regarding oral health.^37^ IDIs will explore access to oral health services, identify existing disparities and provide insights into care-seeking behaviours across the different community settings in the two taluks. To conclude this phase, a Theory of Change workshop will be conducted with key health system stakeholders to collectively map out the causal pathway, identify key activities, assumptions and necessary resources and outline the expected short-term, medium-term and long-term outcomes.^38^ Participants will include representatives from the Indian Council of Medical Research, the DCI, partnering dental colleges and health system personnel such as MOs, THOs, district health officers and dental officers from the respective taluks. This workshop will aim to foster collaboration among stakeholders and facilitate the co-creation of a detailed blueprint for the proposed intervention package.

##### Sample size and sampling strategy

For the oral health literacy survey, a 50% oral health literacy rate was assumed due to the absence of local contextual data and the variability reported in the existing literature. This conservative estimate was chosen because it maximises variance and yields the largest required sample size. With a 5.1% alpha error, a 95% confidence interval, and a design effect of 1.8, the required sample size was 664. To account for a 10% non-response rate, the final sample size will be adjusted to 738. A multi-stage stratified random sampling method will be used, with one taluk selected from each district through purposive sampling.

##### Participant selection

On obtaining informed consent, survey data will be collected from 369 participants each in Kollegal and T. Narasipura taluks using a multi-stage stratified random sampling method, ensuring diversity in age, gender and place of residence.

For qualitative data, IDIs will be conducted with community members selected through snowball sampling, facilitated by community health officers (CHOs). The interviews will continue until data saturation is reached, ensuring a diverse representation of demographics and clinical characteristics. IDIs will be conducted in the local language, with only the participant, research officer and note-taker present.

##### Inclusion criteria and exclusion criteria

The inclusion criteria for the study are participants with a history of dental diseases or dental treatment in the past. Participants must be willing and able to provide relevant information about their dental health history, including previous treatments and current oral health status, and have sought or accessed primary healthcare services within the specified location. The study will include participants from diverse backgrounds, considering factors such as gender, ethnicity and socioeconomic status. Exclusion criteria include participants who choose to withdraw their consent and are unwilling to participate in the IDI, those from outside the geographical area and individuals without any history of dental diseases.

##### Data management and storage

Quantitative data, including oral health literacy and secondary data, will be stored in text format, CSV or PDF files, while the facility preparedness checklist will be saved in CSV or Google Sheets. Qualitative data, such as IDIs, will be stored in Docx format, and workshop data will be maintained in text format. All data, including quantitative, qualitative and media files, will be stored on a password-protected project computer at the project office and backed up via a cloud-based service. Survey data will be entered into Excel, backed up on Google Drive and stored locally in a hard disk. Audio recordings and transcriptions will also be password-protected. Each participant’s information will be anonymised using a unique numerical identifier to maintain confidentiality and protect participant privacy.

##### Data analysis

**Figure 2:**
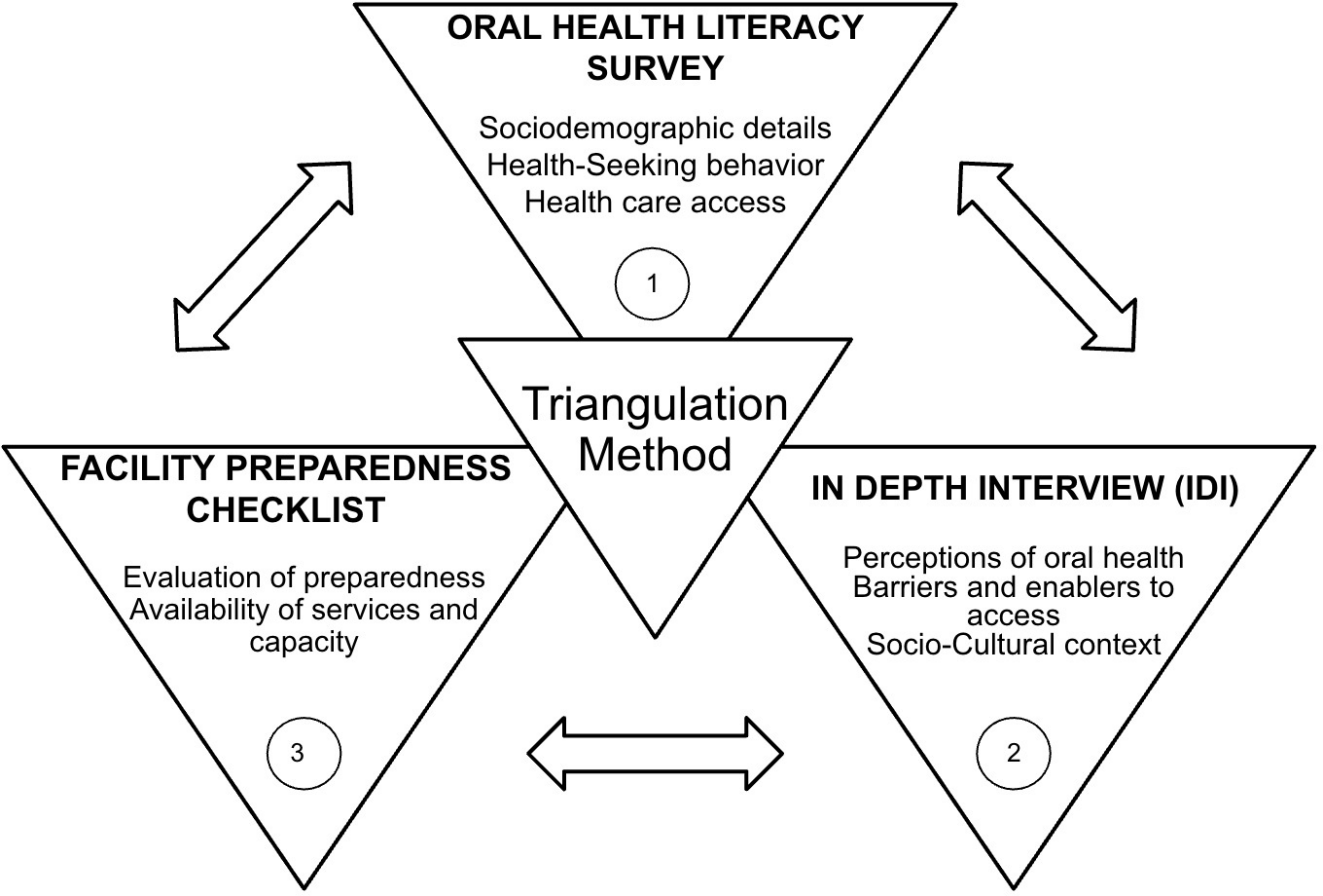
Description of the triangulation method.

Continuous variables from the Oral Health Literacy Survey will be analysed using the mean for normally distributed data and the median for skewed data. Confidence intervals will be calculated, and frequency analysis will assess categorical variables. χ^2^ tests will examine associations, while logistic regression will assess relationships between oral health literacy, access to services and demographic factors (age, gender, socioeconomic status). Compliance scores for the Facility Preparedness Checklist, covering eight themes and 20 subthemes, will be calculated for each PHC, with items scored from 0 to 2 and preparedness expressed as a percentage. Descriptive and comparative analyses will highlight gaps and strengths across facilities. Qualitative data will undergo thematic analysis using an inductive approach to identify key themes. Triangulation will integrate data from the survey, interviews and checklist, providing a comprehensive understanding of oral health access by exploring sociodemographic factors, service preparedness and perceived barriers and enablers (figure 2).

### II. Pilot phase and implementation phase

The second phase involves piloting and implementation: The DHARANI model, adapted from Valentijn *et al*, developed in the formative phase, will be piloted in one PHC in each district to assess its feasibility and identify any implementation challenges, following which it will be implemented in all the PHCs of the block.^36^

The intervention package will include capacity building at the core of the model, with iterative revisions based on stakeholder feedback to develop a context-specific, optimised intervention. Developing Information Education Communication (IEC)/Behaviour Change Communication, which is contextual and in local language for oral health awareness and disease prevention. An implementation support team will closely collaborate with the district health system, establishing routine monitoring mechanisms in the selected block.

**Figure 3:**
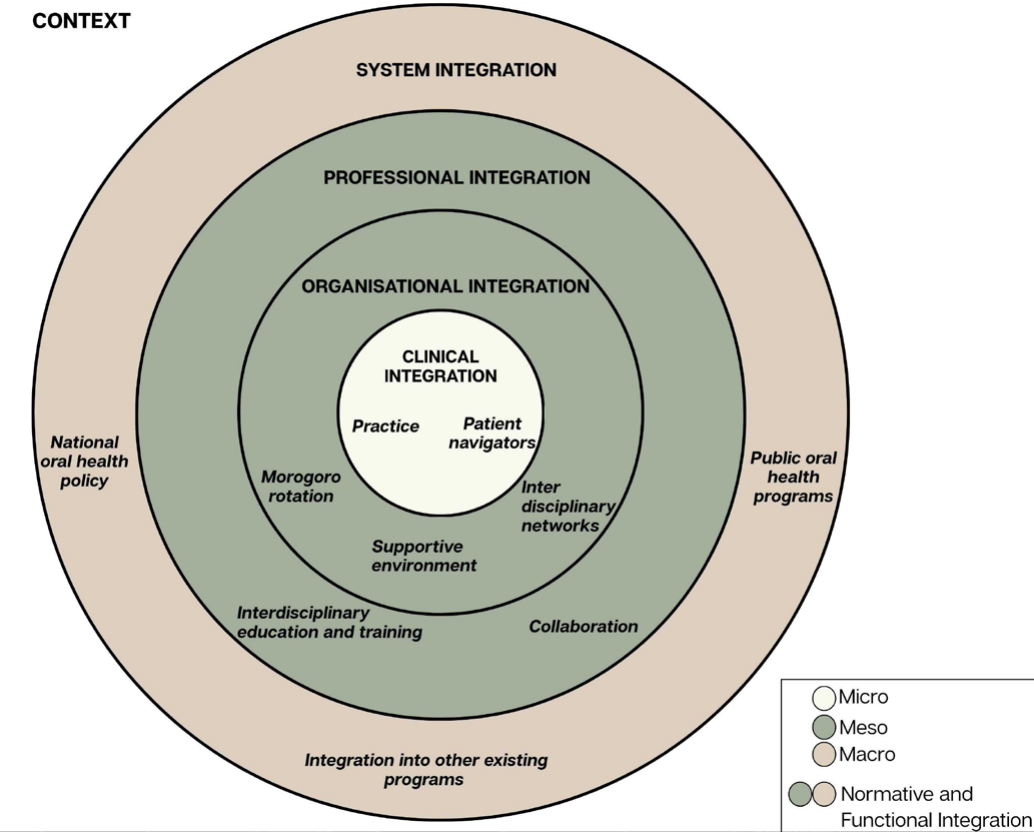
Conceptual framework for integrated oral healthcare based on the integrative functions of primary care.

The conceptual framework, as outlined in figure 3 is grounded in the integrative functions of primary care.^36^ This approach emphasises macro-level, meso-level and micro-level integration to ensure comprehensive, sustainable oral healthcare delivery. The framework also incorporates functional and normative integration to address systemic and cultural factors essential for effective implementation.

#### Macro-level integration

Effective integration of oral health interventions with broader health programmes and policies at the macro level is fundamental for enhancing the delivery and efficiency of systemic healthcare. The National Oral Health Programme (NOHP) is a key initiative aimed at improving oral health outcomes in India. Its objectives include addressing oral health determinants by mitigating risk factors like poor hygiene and inadequate nutrition, reducing morbidity from oral diseases such as dental caries and oral cancers, integrating oral health promotion into the general healthcare system and fostering partnerships to expand reach and impact.^39^ The NOHP operates through a structured hierarchy of national, state and district oral health cells and is implemented through nodal and programme officers at the state and district levels.^40^

One of the strategies for oral health strengthening is the training and capacitation of community health workers already engaged in various health programmes.^20^ By equipping ASHAs, primary healthcare officers and CHOs with oral health knowledge via training modules on oral health awareness, promotion and prevention of oral diseases with diagnosis and referral, we can leverage their established community networks to enhance outreach and service delivery at AAM. The NOHP can significantly benefit from other health programmes such as Rashtriya Bal Swasthya Karyakram (RBSK), the National Program for Prevention and Control of Fluorosis (NPPCF) and the National Tobacco Control Program (NTCP).^3941^,^43^ By moving beyond isolated approaches, integration at the programme levels could enhance their effectiveness. The RBSK, which screens children from birth to 18 years in Anganwadi centres and schools, presents a significant opportunity for integration. Healthcare professionals involved in RBSK could be trained to incorporate oral health promotion into their routine activities. For example, RBSK’s child health screenings could include assessments of oral hygiene, caries and malocclusion, alongside education on proper brushing techniques.^42^ The NPPCF offers an opportunity for incorporating oral health education, as fluorosis, a condition caused by excessive fluoride intake, has notable oral manifestations such as enamel discolouration and fragility. By integrating oral health education into fluorosis prevention efforts, community awareness and early intervention could be improved.^18^ Tobacco use is a major contributor to oral diseases such as oral cancer, periodontal disease and tooth loss. The NTCP, with its mandate to collaborate with other national initiatives, offers a crucial opportunity to address these issues comprehensively. Activities such as training programmes, IEC campaigns, establishing cessation services and working with Panchayati Raj Institutions to promote tobacco control at the grassroots level can complement oral health strategies. This collaborative approach can significantly enhance efforts to reduce the burden of tobacco-related oral health issues.^20^

The integration of oral health interventions into existing national programmes not only ensures resource optimisation but also aligns oral health promotion with broader public health priorities. Such cross-sectoral collaboration addresses systemic gaps and ensures oral health is embedded into the primary healthcare agenda. By strengthening inter-programme coordination, particularly through shared training and community engagement strategies, the healthcare delivery system can address both systemic and specific oral health challenges more effectively.

#### Meso-level integration

Meso-level integration addresses the critical interplay between organisational frameworks and professional capacity-building to improve accessibility, quality and continuity of oral healthcare. This dual approach enables a comprehensive and collaborative effort to embed oral health into the broader healthcare system.

#### Organisational integration

Organisational integration seeks to establish robust partnerships and networks that facilitate a coordinated approach to oral healthcare delivery. A key example is the incorporation of dental colleges into community health initiatives through Morogoro rotation programmes. The Morogoro rotation is a community participation field experience held in rural areas. Students from dental colleges come into the community to conduct camps, offering oral health screenings, education and basic treatments, extending services to underserved populations while fostering experiential learning.^18^

The development of an interdisciplinary network linking dental colleges, dental professionals, district health offices and health system actors is vital for streamlining resources and aligning NOHP’s objectives. These networks promote efficient communication and collaboration, ensuring that oral healthcare interventions are well-integrated into broader health agendas. Such partnerships can provide funding, technical expertise and innovative solutions to expand oral health services.

#### Professional integration

Professional integration focuses on enhancing the skills and competencies of healthcare providers to deliver oral healthcare as part of their routine responsibilities. This requires a systematic approach to capacity-building, encompassing both in-person and virtual training platforms.

Healthcare workers’ in-person, hands-on training sessions at PHCs can equip them with the skills to perform oral health screenings, offer preventive advice and guide patients through referral pathways for advanced care. Online platforms, such as a massive open online course (MOOC), can complement these in-person sessions by offering accessible, self-paced modules on key topics such as oral disease prevention, classification and management. In LMICs, MOOCs play a critical role in addressing gaps in healthcare worker education by providing flexible learning opportunities, which are beneficial for areas with limited access to traditional training infrastructure.^44 45^ These programmes should be tailored to the local context, ensuring cultural and linguistic relevance to maximise their impact.

#### Micro-level integration

At the micro level, the emphasis is on clinical integration, which plays a pivotal role in bridging the gap between primary care and specialised oral healthcare services. This integration begins with community-based oral health initiatives that raise awareness through culturally appropriate communication methods and organising dental diagnosis and treatment camps. These camps also serve as a platform to promote oral hygiene practices and educate the community on the importance of early detection and prevention of oral diseases, including dental caries, periodontal diseases and oral cancers.

Patient navigators are ASHAs from the community to assist in accessing oral health services and navigating referral pathways. This support is particularly valuable for marginalised populations who may encounter linguistic, cultural or logistical barriers in seeking care.

Furthermore, engaging with community stakeholders, such as local leaders and health committees, ensures that clinical integration efforts are tailored to the community’s specific needs. Regular feedback from the community helps refine service delivery, fosters trust and strengthens the relationship between the healthcare system and the population it serves.

#### Functional and normative integration

Effective integration in healthcare systems requires harmonisation at multiple levels. Functional integration refers to the alignment of financial, managerial and informational systems to improve the coordination and efficiency of service delivery. In the context of oral health, functional integration can be achieved through several key mechanisms, such as integrating NOHP’s initiatives at the state level with other programmes and policies to ensure that efforts are supported with alignment that facilitates resource allocation, workforce training and service delivery, reducing duplication and enhancing programme efficiency.

Normative integration focuses on establishing stakeholders’ shared mission, vision and values. It emphasises cultural alignment and collective responsibility for achieving oral health as a component of overall health. Developing a common understanding of the importance of oral health among stakeholders, including NOHP, RBSK and dental colleges, creates a unified framework for action. A shared mission reinforces commitment and promotes intersectoral collaboration. Outreach programmes should emphasise the role of oral health in preventing systemic diseases, such as diabetes and cardiovascular conditions.

By embedding functional and normative integration into oral health programmes, healthcare systems can achieve greater sustainability and impact. These strategies not only strengthen service delivery but also foster a culture of collaboration and inclusivity, ultimately contributing to improved health outcomes at the population level.

This multilevel framework demonstrates a holistic approach to integrating oral health into primary healthcare systems. The focus on capacity building, interdisciplinary collaboration and systemic alignment underpins the potential to transform oral health outcomes in resource-limited settings.

### III. Evaluation phase

This phase will be guided by the Medical Research Council’s framework for the evaluation of complex interventions.^35^ Complex intervention research goes beyond determining whether an intervention meets its intended outcomes. It also investigates its wider impacts, assesses its cost-effectiveness in relation to the resources needed, explores its underlying mechanisms, examines how it interacts with the context in which it is implemented and evaluates its contribution to system change.

**Figure 4:**
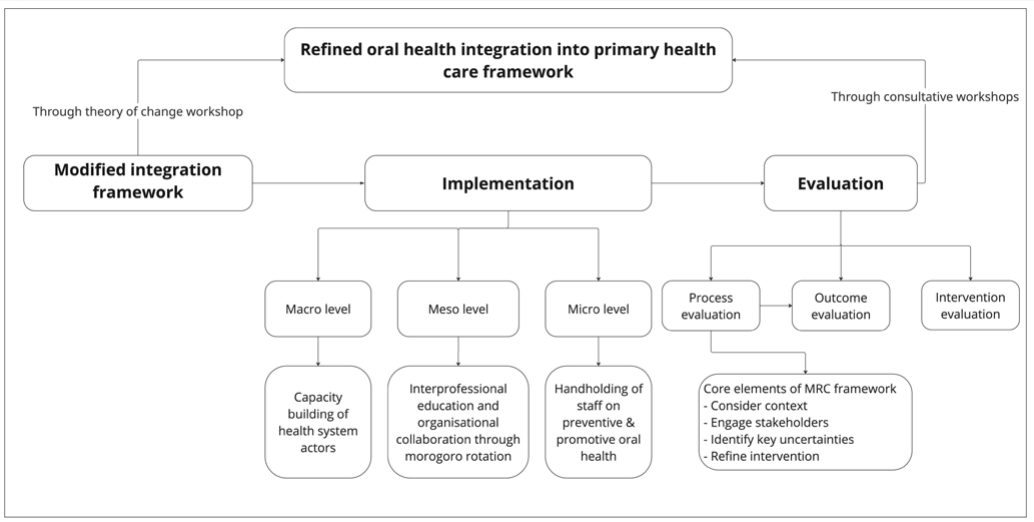
A phased implementation and evaluation framework for integrating oral health into primary healthcare. MRC, Medical Research Council.

Complex intervention research generally includes multiple phases: identifying or developing the intervention, assessing its feasibility and evaluation design, evaluating its effectiveness and ensuring its successful implementation. This study follows the specific adaptations of the MRC framework for developing and evaluating the intervention, as outlined in figure 4.

#### Process evaluation

The framework incorporates a comprehensive context assessment using data collected through IDIs, structured observations and facility checklists to understand the intervention’s environment. Stakeholder engagement is facilitated at multiple levels; macro, meso and micro, using primary research, workshops and interactive sessions. Documentation of these engagements is maintained in a running record. Monthly activity reviews help identify uncertainties and address emerging challenges. The iterative process of refining the intervention focuses on identifying barriers and enablers, which is achieved through document analysis and consultative meetings.

#### Implementation evaluation

The implementation will be measured through its fidelity, feasibility and sustainability. Fidelity is assessed by monitoring adherence to the intervention’s design, while feasibility is evaluated through stakeholders’ feedback and the intervention’s adaptability to different contexts. Data collected via workshops, interviews and routine reporting systems provide critical insights into the intervention’s performance. Sustainability is assessed by examining how well the intervention integrates into existing health systems and its capacity to operate independently over time.

#### Outcome evaluation

The project’s outcomes will be evaluated at three levels: macro, meso and micro. At the macro level, key indicators include the proportion of the population screened or treated at PHCs and the percentage of PHCs offering oral health services assessed via registry reviews. At the meso level, outcomes focus on organisational integration, such as the number of dental staff engaged in community screenings and the frequency of consultative meetings. At the micro level, outcomes emphasise practice-related aspects, including the number of dental camps organised, increased patient visits for dental care and a rise in preventive, curative and rehabilitative oral healthcare needs, tracked through camp records and patient logs. Collectively, these indicators provide a comprehensive evaluation of the integration of oral health services and improved access to care. The study outcome measures are detailed in table 1.

**Table 1:** Study outcome measures

Level	Intervention component	Outcome (what is being measured)	Outcome indicator (name of the measure)
Macro	1. Integration across programmes	Improved care-seeking for oral health	% of individuals who accessed dental care at PHC.
		Strengthened community awareness on oral health	% of VHSNCs discussing oral health in monthly meetings
Meso	1. Interdisciplinary capacity building	Improved capacity for oral health screening and referral	1. % of PHC staff reporting improved confidence to deliver services
			2. Pre-capacity and post-capacity building knowledge assessment
	2. Morogoro rotation	Improved oral health service availability at the PHC-level camps	Proportion of individuals accessing oral health services at PHC-level camps
	3. Stakeholder collaboration	Improved ownership and prioritisation of oral health	% increase in patients availing oral health services at PHCs compared with baseline, based on HMIS data
Micro	1. Community awareness using culturally appropriate IEC materials	Improved awareness of oral health services at the community level	% increase in patients availing oral health services at PHCs (HMIS data) compared with baseline

AAMs, Ayushman Arogya Mandirs; ASHAs, Accredited Social Health Activists; CHOs, Community Health Officers; DCI, Dental Council of India; DHARANI, The Dental Healthcare Access Research and Integration into Primary Healthcare in Karnataka; HMIS, Health Management Information System; IDIs, In-Depth Interviews; IEC, Information Education Communication; LMICs, Low- and Middle-Income Countries; MO, Medical Officer; MOOC, Massive Open Online Course; MRC, Medical Research Council; NOHP, National Oral Health Programme; NPPCF, National Program for Prevention and Control of Fluorosis; NTCP, National Tobacco Control Program; PHC, Primary Health Centre; RBSK, Rashtriya Bal Swasthya Karyakram; THOs, Taluk health officers; VHSNCs, Village Health, Sanitation and Nutrition Committees; WHO, World Health Organization.

## Patient and public involvement statement

Community members’ perspectives will be gathered through oral health literacy surveys and IDIs during the formative phase. A stakeholder consultation workshop will co-develop the Theory of Change, ensuring that community voices and health system actors guide the design of the intervention.

## Ethics and dissemination

This study has received ethical approval from the Institutional Ethics Committee at the Institute of Public Health, Bengaluru (IPH/23-24/E/374). Participants will receive a printed Participant Information Sheet outlining the study’s purpose, procedures, risks and benefits. Informed consent, including permission for audio recording, will be obtained in a language they understand. Participation is voluntary, with no incentives offered, and participants may withdraw at any time without penalty. Data will be anonymised, with personal details restricted to research scientists. All data will be securely stored for 5 years and subsequently destroyed.

The plan for disseminating the results involves conducting a final workshop to present the findings. A critical engagement with state and district health departments, Non-Governmental Organizations and dental colleges will ensure effective communication of the results. Progress will be shared through seminars and presentations at national and international conferences. Key findings will be submitted to peer-reviewed journals for publication.
