# Supplementary material for: Integrating Dental Healthcare into Primary Health Care through an implementation model in two districts of Karnataka in India: An implementation research study protocol
Source: BMJ Open. 2026 Jun 10;16(6):e111996. doi: 10.1136/bmjopen-2025-111996 (PMC13264862; doi:10.1136/bmjopen-2025-111996)
Supplement: Supplementary file 1 [file bmjopen-2025-111996.reviewer_comments.pdf]

PEER REVIEW HISTORY

BMJ Open publishes all reviews undertaken for accepted manuscripts. Reviewers are asked to complete a checklist review form and are provided with free text boxes to elaborate on their assessment. These free text comments are reproduced below.

ARTICLE DETAILS

Title (Provisional)

Integrating Dental Healthcare into Primary Health Care through an implementation model in two districts of Karnataka: An implementation research study protocol

Authors

Chilgod, Lathadevi; Krishna B M, Ramya; Raveendran, Raveena; Hebbar, Pragati

---

VERSION 1 - REVIEW

**Reviewer**	**1**
**Name**	**Ghasemi, Hadi**
**Affiliation**	**Shahid Beheshti University of Medical Sciences, Dept. of Community Oral Health**
**Date**	**17-Oct-2025**
**COI**	**None**

Comments on Integrating Dental Health into Primary Health Care ….

This is an interesting and valuable study addressing key elements of the oral health system. While the manuscript adopts a comprehensive approach, certain aspects would benefit from further elaboration to enhance clarity. I acknowledge that the authors have covered most components of the study protocol; however, to ensure that all elements are fully addressed, I recommend reviewing and completing the attached checklist. Please revisit sections that are already described in the manuscript and expand or clarify any areas that remain insufficiently detailed or unexplained.

Checklist for a Study Protocol on Integrating Dental Care into Primary Health Care

---

1. Title

- Clear, descriptive title (e.g., *Developing an Implementation Model for Integrating Oral Health into Primary Health Care in [City, Country]: A Study Protocol*).

---

2. Abstract

- Structured summary (Background, Aim, Methods, Expected Outcomes, Keywords).
- Briefly define the implementation setting (e.g., urban PHC network, underserved areas).

---

3. Background and Rationale

- Describe **the problem** — burden of oral diseases, fragmentation of care, inequities in access.
- Summarize **evidence gap** — why integration of oral health into PHC is needed.
- Reference global and national frameworks (e.g., WHO 2021 Oral Health Action Plan, Alma-Ata, Astana Declarations).
- Explain the **local context** — health system structure, PHC coverage, and challenges in your study location.
- Identify **previous initiatives** (if any) and their limitations.
- State how this protocol contributes to the literature (e.g., developing a context-specific, replicable model).

---

4. Objectives

- Main aim (e.g., "to develop, implement, and evaluate a model for integrating dental care into PHC").
- Specific objectives:
  - To assess existing oral health services within PHC facilities.
  - To engage stakeholders in co-designing an integration model.
  - To pilot and evaluate feasibility, acceptability, and sustainability.

---

5. Conceptual / Theoretical Framework

- Identify guiding frameworks (e.g., WHO Health System Building Blocks, RE-AIM, Consolidated Framework for Implementation Research (CFIR), or PRECEDE–PROCEED model).
- Describe how the framework guides model development, implementation, and evaluation.
- Provide a conceptual diagram (optional).

---

6. Study Design

- Specify the study type (e.g., mixed-methods, participatory action research, or implementation research).
- Define study phases:
  1. **Situational Analysis / Needs Assessment**
  2. **Model Development (Co-design)**
  3. **Pilot Implementation**
  4. **Evaluation and Refinement**
- Describe how findings from each phase inform the next.

---

7. Study Setting

- Describe the PHC network (number and type of centers, workforce, catchment area).
- Define geographic, demographic, and socioeconomic characteristics of the target population.
- Explain why the setting is suitable for integration.

---

8. Stakeholder Involvement and Community Engagement

- Identify stakeholders: PHC providers, dentists, health managers, policymakers, community representatives.
- Describe the stakeholder engagement process (focus groups, workshops, advisory panels).
- Mention how community input will shape model components and ensure social acceptability.

---

9. Components of the Integration Model

Use the **TIDieR framework** (Template for Intervention Description and Replication):

Element	Description
**Why**	Rationale for each component (e.g., oral health screening within PHC visits).

Element	Description
**What**	Activities/interventions (training, equipment, referral protocols, health education).
**Who**	Responsible personnel (PHC physicians, nurses, dentists, community health workers).
**How**	Mode of delivery (face-to-face training, electronic referral, community outreach).
**Where**	PHC centers, schools, community spaces.
**When/How much**	Duration, frequency, intensity.
**Tailoring**	How model is adapted to local context.
**Modifications**	Plans for iterative improvement during pilot.
**Implementation fidelity**	How adherence to model will be monitored.

---

10. Data Collection and Analysis

Quantitative components:

- Surveys, service utilization records, referral data, oral health indicators.
- Pre- and post-implementation comparisons (e.g., % of PHC clients screened for oral conditions).
- Statistical methods for descriptive and inferential analysis.

Qualitative components:

- Interviews or focus groups with stakeholders.
- Thematic analysis to capture barriers, facilitators, perceptions of feasibility and acceptability.

Process evaluation:

- Indicators based on the RE-AIM framework (Reach, Effectiveness, Adoption, Implementation, Maintenance).

---

11. Outcome Measures

Define measurable indicators under three domains:

Domain	Indicators / Examples
**Implementation outcomes**	Feasibility, fidelity, acceptability, coverage.
**Service delivery outcomes**	Number of oral health screenings, referrals completed, preventive interventions delivered.

Domain	Indicators / Examples
**Health outcomes**	Oral hygiene status, DMFT index, patient satisfaction, oral health literacy.

---

12. Ethical Considerations

- Approval from institutional ethics committee.
- Informed consent procedures for participants.
- Data privacy and confidentiality.
- Risk–benefit assessment.

---

13. Project Management and Timeline

- Describe project phases, timeline (e.g., Gantt chart).
- Mention team roles (PI, co-investigators, field coordinators).

---

14. Dissemination and Knowledge Translation

- Plans for sharing findings with stakeholders, health authorities, and the community.
- Plans for publication and presentation.
- Strategy for embedding the model into local or national health systems.

---

15. Strengths and Limitations

- Anticipated challenges (e.g., resource constraints, staff turnover).
- Potential biases (selection, response, social desirability).
- Strategies to mitigate these limitations.

---

16. Funding and Conflict of Interest

- Funding source and grant number (if applicable).
- Declaration of no conflict of interest if applicable.

---

17. References and Supplementary Material

- Include key literature (WHO 2021, national health policy, previous integration models).
- Attach any data collection tools, frameworks, or figures as supplementary files.

**** **The reviewer provided a marked copy with additional comments. Please contact the publisher for full details.** ****

**Reviewer**	**2**
**Name**	**Khami, Mohammad Reza**
**Affiliation**	**Tehran University of Medical Sciences, Tehran**
**Date**	**16-Feb-2026**
**COI**	**None**

This is a to-the-point study on an important subject. There are certain points that should be addressed:

Introduction:

Page 3, line 36: The full term for ANMs, ASHAs is needed.

Page 4, lines 12 and 13: The sentence seems to be incomplete.

Page 4, line 37: To build…

In the Introduction, the health care system of India, at least in the two districts of interest should be briefly explained.

Too many references in the Introduction have been used. Please limit to most recent and most valid ones.

Methods:

An important group of stakeholders is the health care personnel (including dental professionals) working in the PHC centers. How they would be involved? Are they included in the interviews? This is also true for lay people, and their views should be explored. For example: what do they expect from the PHC system regarding oral health?

What does Morgoro rotation mean in Figure 3? Please add in footnote.

The first paragraph of page 10 can be moved to Discussion part. I suggest adding a short Discussion section with this and similar paragraphs.

Are the interventions already determined? If yes, it would be good to add a table or figure to show them at a glance. If not, and they would be fully determined after the first phase, then this should be elaborated and explained.

Page 12, line 10: …implemented, and…

In Figure 4, please write the full term of MRC, as each table and figure should be self-standing.

Reference 40 is incomplete.

The WHO Global Oral Health Action Plan 2023-2030 can be added as reference.

VERSION 1 - AUTHOR RESPONSE

Reviewer: 1
Dr. Hadi Ghasemi, Shahid Beheshti University of Medical Sciences

Comments to the Author:
See attached files.

Attached file:
Comments on Integrating Dental Health into Primary Health Care

This is an interesting and valuable study addressing key elements of the oral health system. While the manuscript adopts a comprehensive approach, certain aspects would benefit from further elaboration to enhance clarity. I acknowledge that the authors have covered most components of the study protocol; however, to ensure that all elements are fully addressed, I recommend reviewing and completing the attached checklist. Please revisit sections that are already described in the manuscript and expand or clarify any areas that remain insufficiently detailed or unexplained.

Checklist for a Study Protocol on Integrating Dental Care into Primary Health Care

1. Title

Author response:
The title clearly reflects the scope, setting, and design of the study.

> "Integrating Dental Healthcare into Primary Health Care through an implementation model in two districts of Karnataka: An implementation research study protocol."

2. Abstract

Author response:
The abstract describes the study design, setting, and objectives in full sentences.

> “A mixed-methods implementation research approach will be adopted for this study, comprising three phases: formative, pilot & implementation, and evaluation.”

> “This study aims to develop an implementation model for integrating dental health care into primary health care systems in Chamarajanagar and Mysuru districts of Karnataka.”

> “A mixed-methods study at the district level will be conducted to capture diverse perspectives and characterise inequalities in oral health care access and literacy levels across urban, rural, and tribal areas.”

3. Background and Rationale

Author response:
The manuscript describes the burden of oral diseases and inequities in access.

> “Oral health is a major global health concern, with 45% of the world's population affected by dental diseases, particularly in low- and middle-income countries (LMICs). In India, where up to 75% of the population suffers from dental diseases, several challenges exacerbate the situation, which includes inadequate healthcare-seeking behaviours, insufficient national data on oral health indicators, a shortage of public sector dentists, and the dominance of private dental services with high out-of-pocket expenses.”

It further describes inequities and system gaps.

> “The limited availability of public sector dentists and an inequitable allocation of resources in urban areas results in significant gaps in oral healthcare delivery. The high costs of private dental services render treatment inaccessible for a substantial segment of the population, particularly those residing in underserved areas.”

The need for integration is articulated.

> “Addressing these challenges of oral health inequities requires innovative solutions, including the adoption of integrated models of care which offer insights into improving oral healthcare accessibility.”

Global and national frameworks are referenced.

> “One of the six guiding principles of the Global strategy on oral health also emphasises incorporating oral health into primary healthcare alongside life course-specific interventions and leveraging digital technologies for oral health.”

> “The Government of India has made efforts to address disparities in oral healthcare through initiatives like the National Oral Health Policy and the incorporation of basic oral care services within Ayushman Arogya Mandirs.”

The local context is described.

> “India’s public healthcare system follows a three-tier hierarchy of primary, secondary, and tertiary care. In Karnataka, this is delivered through Primary Health Centres (PHCs), Community Health Centres (CHCs), and District Hospitals. At the grassroots level of this hierarchy are the Ayushman Arogya Mandirs (AAMs), which serve as the primary care points for providing comprehensive services to the community.”

The gap in implementation is identified.

> “Despite the success of these models elsewhere, progress in developing and implementing such frameworks within India has been limited.”

4. Objectives

Author response:
The objectives are stated in the manuscript as follows.

> “The research has three objectives:
> To characterise inequalities and describe oral health care-seeking practices among heterogeneous communities residing in urban, rural, and tribal areas of two districts of southern India.
> To co-design and pilot integrated primary and oral health intervention with health system actors.
> To build an implementation model for an integrated oral health solution at the district level.”

5. Conceptual / Theoretical Framework

Author response:
The study framework is described using established models.

> “The overarching framework for the study is based on the Medical Research Council (MRC) guidance for developing and evaluating complex interventions.”

> “To guide the integration process, the study adopts a modified version of Valentijn et al.'s integration model that supports the alignment and coordination of healthcare efforts across multiple system levels.”

The multilevel nature of the framework is explained.

> “This approach emphasizes macro, meso, and micro-level integration to ensure comprehensive, sustainable oral healthcare delivery. The framework also incorporates functional and normative integration to address systemic and cultural factors essential for effective implementation.”

6. Study Design

Author response:
The study design and phases are clearly described.

> “This study employs an Implementation Research (IR) approach, using mixed-methods organised into three key phases: (I) the Formative Phase, (II) the Pilot and Implementation Phase, and (III) the Evaluation Phase.”

7. Study Setting

Author response:
The study setting and population are described.

> “The study will take place in two districts of southern Karnataka, India: Chamarajanagar and Mysuru. Within these districts, one block from each, Kollegal taluk in Chamarajanagar and T. Narasipura taluk in Mysuru will be purposefully chosen.”

> “The study districts, Chamarajanagar and Mysuru, encompass diverse urban, rural, and tribal populations.”

8. Stakeholder Involvement and Community Engagement

Author response:
The manuscript describes stakeholder engagement processes.

> “Participants will include representatives from the Indian Council of Medical Research (ICMR), the Dental Council of India (DCI), partnering dental colleges, and health system personnel such as Medical Officers (MOs), Taluk Health Officers (THOs), District Health Officers (DHOs), and dental officers from the respective taluks.”

> “To conclude this phase, a Theory of Change workshop will be conducted with key health system stakeholders to collectively map out the causal pathway.”

Community engagement is included.

> “survey data will be collected ensuring diversity in age, gender, and place of residence, in-depth interviews (IDIs) will be conducted with community members.”

9. Components of the Integration Model

Author response:
The intervention components are described in detail.

> "The intervention package will include capacity building at the core of the model, with iterative revisions based on stakeholder feedback to develop a context-specific, optimized intervention. Developing Information Education Communication/Behaviour Change Communication (IEC/BCC), which is contextual and in local language for oral health awareness and disease prevention. An implementation support team will closely collaborate with the district health system, establishing routine monitoring mechanisms in the selected block."

Modes of delivery and activities are also described.

> "Healthcare workers' in-person, hands-on training sessions at PHCs can equip them with the skills to perform oral health screenings. Online platforms, such as a massive open online course (MOOC) can complement these in-person sessions."

> "This integration begins with community-based oral health initiatives that raise awareness, organising dental diagnosis and treatment camps."

10. Data Collection and Analysis

Author response:
The manuscript details quantitative and qualitative methods.

> "Continuous variables from the Oral Health Literacy Survey will be analysed using the mean for normally distributed data and the median for skewed data. Chi-square tests will examine associations, while logistic regression will assess relationships."

> "Qualitative data will undergo thematic analysis using an inductive approach to identify key themes."

Integration of findings is described.

> "Triangulation will integrate data from the survey, interviews, and checklist, providing a comprehensive understanding of oral health access."

11. Outcome Measures

Author response:
Outcome measures are described across levels.

> “At the macro level, key indicators include the proportion of the population screened or treated at PHCs and the percentage of PHCs offering oral health services.”

> “At the meso level, outcomes focus on organisational integration, the number of dental staff engaged in community screenings and the frequency of consultative meetings.”

> “At the micro level, outcomes emphasise practice-related aspects, including the number of dental camps organised, increased patient visits for dental care, and a rise in preventive, curative, and rehabilitative oral healthcare needs.”

12. Ethical Considerations

Author response:
Ethical procedures are described.

> “This study has received ethical approval from the Institutional Ethics Committee at the Institute of Public Health, Bengaluru (IPH/23-24/E/374). Participants will receive a printed Participant Information Sheet outlining the study’s purpose, procedures, risks, and benefits. Informed consent will be obtained in a language they understand. Participation is voluntary; participants may withdraw at any time without penalty.”

> “Data will be anonymised. All data will be securely stored.”

13. Project Management and Timeline

Author response:
The manuscript describes roles and responsibilities.

> “LC and PH are involved in the conceptualisation of the study. PH is responsible for funding acquisition and overall supervision. LC is responsible for project administration. LC, RK, and RR are involved in writing original drafts. PH is involved in reviewing and editing drafts14. Dissemination and Knowledge Translation

Timeline:The project follows a structured three-phase approach, beginning with a formative phase to triangulate surveys, interviews, and facility checklists into a co-created integration blueprint. This leads into an implementation phase involving MOOC-based and hands on capacity building and camps through dental college rotations, followed by an evaluative phase that uses the MRC framework to assess service utilization and scalability.

Author response:
Plans for dissemination are described.

> "The plan for disseminating the results involves conducting a final workshop to present the findings. Progress will be shared through seminars and presentations at national and international conferences. Key findings will be submitted to peer-reviewed journals for publication."

15. Strengths and Limitations

Author response:
Strengths and limitations are explicitly stated.

> "This study characterises oral health inequalities across urban, rural, and tribal communities. The co-design, pilot, and implementation of an integration model, ensures the approach is context-specific, participatory, and sustainable."

> "The study may be subject to variability in the real-world settings. Data quality is dependent on the accuracy and consistency of records maintained at primary healthcare centres."

16. Funding and Conflict of Interest

Author response:
Funding and conflicts are declared.

> "This research study is supported by the Indian Council of Medical Research(ICMR project ID: IIRPIG-2023-0001466)."

> "None declared."

17. References and Supplementary Material

Reviewer comment:
Include relevant literature and supplementary materials.

Author response:
The manuscript includes references to:

> "WHO reports, national programmes, and integration models."

Figures included:

> "Figure 1 (map), Figure 2 (triangulation), Figure 3 (conceptual framework), Figure 4 (MRC framework)."

Reviewer: 2
Dr. Mohammad Reza Khami, Tehran University of Medical Sciences, Tehran

Comments to the Author:
This is a to-the-point study on an important subject. There are certain points that should be addressed:

Introduction:
Page 3, line 36: The full term for ANMs, ASHAs is needed.
We thank the reviewer for this correction. We have added the full expansions (in page no 2, line number 72 of the manuscript), for ANMs (Auxiliary Nurse Midwives) and ASHAs (Accredited Social Health Activists) in the revised manuscript.

Page 4, lines 12 and 13: The sentence seems to be incomplete.
Thank you for identifying this grammatical error. We have revised the text in page number 3, line 100 of the manuscript to ensure the sentence is complete.
Revised text: *"While some studies underscore the challenges and solutions in integrating oral health into primary care, focusing on enhancing health outcomes and reducing disparities, they emphasize that success depends heavily on structured training for non-dental providers and the establishment of clear referral pathways(46,47). Key enablers include.."*

Page 4, line 37: To build…
Thank you. We have rephrased the third objective as follows in page number 3, line 137 of the manuscript:
"3. To build an implementation model for an integrated oral health solution at the district level".

In the Introduction, the health care system of India, at least in the two districts of interest should be briefly explained.
Thank you for this suggestion, as it provides essential context for the study's implementation setting. We have added a concise description of the Indian healthcare system to the Introduction in page number 3, line 113 of the manuscript
Revised introduction: "The public healthcare system in India is structured as a three-tier hierarchy designed to deliver primary, secondary, and tertiary care, operating alongside an extensive private sector. Primary care is the first point of contact, delivered through a network of Ayushman Arogya Mandirs (AAMs) and Primary Health Centres (PHCs), former being the grassroot level centre for the community and the latter typically led by an administrative Medical Officer. Secondary care is provided through Community Health

Centres (CHCs) and Taluk Hospitals led by Taluk health officers, while tertiary care is managed via District Hospitals and Medical Colleges. The Dental Healthcare Access Research and Integration into Primary Health Care in Karnataka (DHARANI) study is situated within this framework across two districts in Karnataka: Mysuru (specifically T. Narasipura taluk) and Chamarajanagar (Kollegala taluk). These taluks represent a diverse demographic mix of urban, rural, and tribal populations, served by a total of 22 PHCs (14 in T. Narasipura and 8 in Kollegala) along with their constituent AAMs. Despite this infrastructure, oral health services remain largely 'dentist-centered' and concentrated in urban private hospitals, leaving the primary level, with significant service gaps. The DHARANI study aims to develop and pilot an implementation model for integrating equitable dental health care into primary health care in collaboration with health system actors in these two districts (Mysuru and Chamarajanagar) of Karnataka*".*

Too many references in the Introduction have been used. Please limit to the most recent and most valid ones.

We agree with the reviewer's assessment. We have streamlined the Introduction by removing several older and redundant citations. We have prioritized the most recent and valid literature relevant to our implementation model, including the WHO Global Oral Health Action Plan 2023–2030, to ensure the rationale is both evidence-based and concise. The total number of references in the Introduction has been reduced accordingly.

Methods:
An important group of stakeholders is the health care personnel (including dental professionals) working in the PHC centers. How would they be involved? Are they included in the interviews? This is also true for lay people, and their views should be explored. For example: what do they expect from the PHC system regarding oral health?

Thank you for this query. There are no dental professionals working in the PHCs. There are dentists working at CHCs and higher referral centres.
A. We agree that **health care personnel** are an important group of stakeholders and hence they have been involved in the following ways:

- **Co-design:** Medical Officers, District/Taluk Health Officers, Dental and Divisional Officers participate in Theory of Change (ToC) workshops to co-create the integration model.
- **Implementation:** Frontline workers, Community health officers (CHOs) and ASHAs receive capacity building to provide screenings and right referral pathways.
- **Feedback:** Their perspectives on system barriers and training satisfaction are gathered through consultative meetings and workshops.

B. Laypeople/community involvement:

- **Expectations & Barriers:** Community members participate in In-depth Interviews (IDIs) to explore their care-seeking behaviors and expectations of the PHC system.
- **Needs Assessment:** An oral health survey of 738 participants quantifies oral health literacy and community needs to ensure the intervention is context-specific.

What does Morgoro rotation mean in Figure 3? Please add in footnote.

Thank you for the question. As defined in the manuscript under section 'Organisational Integration' at Meso-level integration, the Morogoro rotation is a community participation field experience held in rural areas. Students from dental colleges come into the community to conduct camps, offering oral health screenings, education, and basic treatments, extending services to underserved populations while fostering experiential learning. In the context of Figure 3, it represents the mechanism for Organisational Integration, where academic dental institutions provide the necessary manpower for community screenings and basic care at the PHC level. The following footnote has been added in page 6.

"**Note:** The Morogoro rotation refers to a community participation field experience held in rural areas. Students from dental colleges come into the community to conduct camps, where academic dental institutions provide the necessary workforce for community screenings and basic care at the PHC level."

The first paragraph of page 10 can be moved to the Discussion part. I suggest adding a short Discussion section with this and similar paragraphs.
Thank you for this suggestion. As this is a study protocol paper and the intervention has not yet been fully executed, we have intentionally omitted a formal 'Discussion' section. Instead, we have focused on providing a highly detailed Study Design section that explains the core components of the intervention and its systematic alignment with the MRC framework. We believe that keeping this descriptive detail within the methodology provides a clearer blueprint for the integration process, which is the primary objective of a protocol.

Are the interventions already determined? If yes, it would be good to add a table or figure to show them at a glance. If not, and they would be fully determined after the first phase, then this should be elaborated and explained.

The pre-determined components of the intervention have been shown as in the table below in the section 'outcome evaluation'in page 10 line number 428, as follows:

**Level**	**Intervention component**	**Outcome (What is being measured)**	**Outcome Indicator (Name of the measure)**

**Macro**	1. Integration across programmes	Improved care-seeking for oral health.	% of individuals who accessed dental care at PHC.
		Strengthened community awareness on oral health.	% of VHSNCs discussing oral health in monthly meetings.
**Meso**	1. Inter disciplinary Capacity building	Improved capacity for oral health screening and referral.	1. % of PHC staff reporting improved confidence to deliver services.
			2. Pre- and post-capacity building knowledge assessment.
	2. Morogoro rotation	Improved oral health service availability at the PHC level camps	Proportion of individuals accessing oral health services at PHC-level camps.
	3. Stakeholder collaboration	Improved ownership and prioritisation of oral health.	% increase in patients availing oral services at PHCs (HMIS data) compared to baseline.
**Micro**	1. Community awareness using culturally appropriate IEC materials	Improved awareness of oral health services at the community level.	% increase in patients availing oral services at PHCs (HMIS data compared to baseline).

Table 1: Study outcome measures

Page 12, line 10: implemented, and…

Thank you. This correction has been made on page 9 line number 392.

In Figure 4, please write the full term of MRC, as each table and figure should be self-standing.

Thank you. The full form has been added in page 4 line number 151.

Reference 40 is incomplete.

Thank you for pointing out this incomplete referencing. Since this is an older paper (2014) we have discarded this citation, as per your previous comment.

The WHO Global Oral Health Action Plan 2023-2030 can be added as reference,

Thank you for this suggestion. We have added the relevant reference from WHO Action plan document into our introduction in page 2 line number 84:

“..interprofessional collaborative practice in primary care settings to address structural barriers and develop interoperable infrastructures(39,40). Such approaches are now central to the current WHO global strategy for decentralizing dental services through the primary health care workforce”.

Reviewer: 1
Competing interests: not applicable

Reviewer: 2
Competing interests: -
